# Subthalamic theta activity: a novel human subcortical biomarker for obsessive compulsive disorder

**DOI:** 10.1038/s41398-018-0165-z

**Published:** 2018-06-18

**Authors:** Pnina Rappel, Odeya Marmor, Atira S Bick, David Arkadir, Eduard Linetsky, Anna Castrioto, Idit Tamir, Sara A. Freedman, Tomer Mevorach, Moran Gilad, Hagai Bergman, Zvi Israel, Renana Eitan

**Affiliations:** 1 Department of Medical Neurobiology (Physiology), Institute of Medical Research – Israel-Canada, the Hebrew University-Hadassah Medical School, Jerusalem, Israel; 2 The Edmond and Lily Safra Center for Brain Research, the Hebrew University, Jerusalem, Israel; 3 The Brain Division, Hadassah–Hebrew University Medical Center, Jerusalem, Israel; 4 Grenoble Institute of Neuroscience, Grenoble, France; 5 The Center for Functional and Restorative Neurosurgery, Hadassah-Hebrew University Medical Center, Jerusalem, Israel; 6 Department of Neurosurgery, University of California San Francisco, San Francisco, CA USA; 7 School of Social Work, Bar Ilan University, Ramat Gan, Israel; 8 Department of Psychiatry, Functional Neuroimaging Laboratory, Brigham and Women’s Hospital, Harvard Medical School, Boston, MA USA

## Abstract

Obsessive-compulsive disorder (OCD) is a common and serious psychiatric disorder. Although subthalamic nucleus deep brain stimulation (DBS) has been studied as a treatment for OCD patients the underlying mechanism of this treatment and the optimal method of stimulation are unknown. To study the neural basis of subthalamic nucleus DBS in OCD patients we used a novel, implantable DBS system with long-term local field potential sensing capability. We focus our analysis on two patients with OCD who experienced severe treatment-resistant symptoms and were implanted with subthalamic nucleus DBS systems. We studied them for a year at rest and during provocation of OCD symptoms (46 recording sessions) and compared them to four Parkinson’s disease (PD) patients implanted with subthalamic nucleus DBS systems (69 recording sessions). We show that the dorsal (motor) area of the subthalamic nucleus in OCD patients displays a beta (25–35 Hz) oscillatory activity similar to PD patients whereas the ventral (limbic-cognitive) area of the subthalamic nucleus displays distinct theta (6.5–8 Hz) oscillatory activity only in OCD patients. The subthalamic nucleus theta oscillatory activity decreases with provocation of OCD symptoms and is inversely correlated with symptoms severity over time. We conclude that beta oscillations at the dorsal subthalamic nucleus in OCD patients challenge their pathophysiologic association with movement disorders. Furthermore, theta oscillations at the ventral subthalamic nucleus in OCD patients suggest a new physiological target for OCD therapy as well as a promising input signal for future emotional-cognitive closed-loop DBS.

## Introduction

Obsessive compulsive disorder (OCD) is a debilitating psychiatric disorder characterized by obsessions and compulsions. Although understanding of the etiology of OCD is limited, functional and structural brain imaging studies have revealed aberrant cortico-striato-thalamo-cortical (CSTC) circuits in OCD^1,2^. The subthalamic nucleus (STN) has a central position within the CSTC circuits and through its sensorimotor, emotional and cognitive areas coordinates decision-making, action-selection and various high-order processes^3–6^. STN deep brain stimulation (DBS) has been studied as a treatment for severe resistant OCD patients^6–13^. DBS surgery allows both acute and chronic electrophysiological recordings from DBS targets.

Synchronized oscillatory activity is a feature of healthy brain that facilitates communication between nearby and distant neuronal networks^14^. Pathological variations in oscillatory activity may represent brain disorders. In Parkinson’s disease, excessive synchronized low (12–25 Hz) and high (25–35 Hz) beta band activity in the dorsal STN has been identified as pathognomonic and thought to be responsible for some of the parkinsonian symptoms such as rigidity and akinesia^15–17^. Therapeutic STN DBS reversibly reduces both this pathologic activity and parkinsonian motor symptoms^18^.

Although many studies have investigated the sub-regions of the STN in PD patients, only two groups have reported acute STN recordings in OCD patients^19–23^. These invasive human recordings in a small group of patients have been recorded with intraoperative leads or temporarily externalized leads in the immediate post-operative period. Nonspecific oscillations have been reported in OCD but the relationship to obsessions and compulsions was not clear mainly because OCD symptoms change over time, have specific triggers related to location, timing or situation and are typically reduced immediately following the insertional effects of DBS surgery. To circumvent these difficulties, we used an investigational device, activa PC+S (Medtronic Inc.), that in addition to standard stimulating capabilities, can also record long-term local field potentials (LFPs). This system allows long term functional electrophysiological recordings of the STN throughout different phases of the disease, behavioral challenges and DBS treatment.

## Methods

### Study participants

In this study, two OCD patients and a control group of four PD patients underwent STN DBS surgery with implantation of the Activa PC+S pacemaker (Medtronic, Inc, Minneapolis, MN, USA). All patients met accepted inclusion criteria for DBS surgery and signed informed consent. PD patients had (i) advanced idiopathic PD; (ii) long-term levodopa use with reduced efficacy, on-off motor fluctuations and increased incidence of medication-induced side effects; (iii) normal cognitive function or mild-moderate cognitive decline as defined by Addenbrooke’s cognitive examination (ACE) >75 and frontal assessment battery (FAB) >10. OCD patients were diagnosed by Structured Clinical Interview for DSM-IV (SCID-IV) and had (i) disabling severe symptoms, as assessed by a Yale-Brown Obsessive Compulsive Scale (Y-BOCS) score of 25 or more; (ii) documented highly treatment refractory OCD, including at least three adequate trials of different serotonin reuptake inhibitors (e.g., fluoxetine, sertraline, fluvoxamine, paroxetine, citalopram, escitalopram, and clomipramine) for at least 3 months at the maximum tolerated dose; augmentation of one of the SSRIs with clomipramine, a neuroleptic, or clonazepam (each for at least 2 weeks); and adequate behavior therapy (as indicated by a cognitive behavior therapist with substantial expertize in OCD treatment); (iii) drug-free or on a stable drug regimen for at least 6 weeks before study entry. Patients’ demographics, baseline treatment and optimal stimulation parameters are presented in Table 1. All six patients (four PD patients and two OCD patients) are included in the intra-operative and post-operative analysis (Fig. 2). The second OCD patient declined post-operative follow-up after seven recording sessions. Therefore, only five patients (four PD patients and one OCD patient) are included in the analysis presented in Figs. 3 and 4. This study was authorized and supervised by the IRB of Hadassah Medical Center (no. 0403-13-HMO) and the Israel Ministry of Health (no. HT6752). Clinical Trials Registration number: NCT01962194

**Table 1:** Patients’ demographics, baseline treatment, optimal stimulation parameters and number of recording sessions

Pts.	Group	Age	Gender	Duration of disease (years)	Baseline medications (dose)	Baseline UPDRS III motor score (PD)	Baseline Y-BOCS score (OCD)	Electrode location [*x*, *y*, *z*]	Optimal stimulation parameters left/right: (Frequency (Hz); pulse duration (μs); contact configuration; voltage (V))	Number of recording sessions
Jur 01	PD	66	F	8	Stalevo 50 mg q4d Rasagiline 2 mg qid	35	–	Left: (−12, −4, −4.5) Right: (12.25, −3.5, −5.5)	Left: (180; 60; c + 9−; 2) Right: (180; 60; c + 1−; 1.9)	15
Jur 02	OCD	50	F	35	Clomipramine 75 mg bid Olanzapine 5 mg bid	–	34	Left: (−10.65, −1.5, −5) Right: (10.5, −1, −5.5)	Left: (130; 60; c + 9−; 1) Right: (130; 60; c + 1−; 1)	39
Jur 03	PD	54	M	9	Carbidopa 12.5 mg q3h Levodopa 125 mg q3h Biperiden 1 mg q4h Ropinirole 4 mg qid	31	–	Left: (−11.25, −1.75, −4.5) Right: (11, −2.5, −5)	Left: (130; 60; c + 9−; 2.1) Right: (130; 60; c + 1−; 1.6)	15
Jur 04	OCD	34	M	20	–	–	36	Left: (−11.25, −2.4, −5.25) Right: (11.25, −4, −4)	Left: (120; 60; c + 8−; 0.5) Right: (120; 60; c + 0−; 0.5)	7^a^
Jur 05	PD	52	F	10	Carbidopa 25 mg q6h Levodopa 250 mg q6h	42	–	Left: (−10.5, −2.5, −4) Right: (10.75, −3.5, −5)	Left: (130; 60; c + 8–11−; 1.9) Right: (130; 60; c + 1–2−; 1.3)	22
Jur 06	PD	52	F	9	Carbidopa 25 mg q5h Levodopa 250 mg q5h Rasagiline 1 mg qid Ropinirole 8 mg qid	43	–	Left: (−11, −3, −4) Right: (11.5, −3, −4.5)	Left: (130; 60; c + 9–11−; 2.2) Right: (130; 60; c + 1–3−; 1.8)	17

^a^This OCD patient declined post-operative follow-up after seven recording sessions

### Surgery planning and electrophysiological navigation

The deep brain target chosen in this study for OCD patients was the assumed border between the associative and limbic areas of the STN^17^. This was 2 mm anterior to and 1 mm medial to the target that is used for PD patients^17^. Surgical technique has been previously described^24^. Briefly, target was chosen before surgery according to high resolution T2 weighted MR images and trajectory planning was carried out according to T1 + Gadolinium MR images that were fused with high resolution CT acquired in the morning of surgery. During the surgery, the boundaries and the subdomains of the target nucleus were verified by microelectrode electrophysiological recordings of multi-unit spiking activity along the trajectory. Intra-operative analysis of the microelectrode spontaneous spiking activity recordings along the surgical trajectory, response of spiking activity to passive and active movements of the contralateral limbs and detecting the clinical effects of stimulation at the target assisted with the final localization of the permanent DBS electrode. Intra-operative recordings include multi-unit activity without spike sorting, so no analysis of discharge rate/pattern of single units can be made.

### Intra-operative electrophysiological recordings

In each STN trajectory two electrodes, separated by 2 mm each, were advanced simultaneously in the vertical dimension. The typical distance between vertical recording sites before the entrance to the STN was 200–400 µm. The typical distance between recording sites within the STN was 100 µm. Areas with smaller number of recording sites were postoperatively interpolated to the general number of recording sites. Neurophysiological activity was recorded via polyamide coated tungsten microelectrodes (AlphaOmega Engineering, Nazareth, Israel) with impedance of around of 0.3–0.7 MΩ (measured at 1 KHz). The data were acquired with the NeuroOmega system (AlphaOmega Engineering, Nazareth, Israel). Analysis of intra-operative data had been previously described^24^. The signal was amplified by 20, band-passed from 0.7 to 9000 Hz using a hardware four-pole Butterworth filter, and sampled at 44 kHz by a 16-bit A/D converter (using ±1.25 V input range).

### Post-operative clinical assessment and electrophysiological recordings

Electrophysiological post-operative LFPs (local field potentials) recordings were acquired in an outpatient setting. Patients came for clinical evaluation and recording sessions every 2–4 weeks for a period of 6–12 months. In every recording session, clinical assessments by a psychiatrist (RE) and neurologist (DA and EL) also took place. These included formal psychiatric assessments, including the Yale-Brown Obsessive-Compulsive Scale (Y-BOCS).

The implanted brain leads (model 3389; Medtronic, Inc., Minneapolis, MN) consisted of four contacts, each with a diameter of 1.27 mm and length of 1.5 mm spaced by 0.5 mm intervals. The lead was placed along the dorso-ventral axis of the STN, and contacts were numbered from 0 (ventral) to 3 (dorsal). We used bipolar macroelectrode recordings inside the STN. This method has been shown to overcome volume conductance effects and reflect locally generated neuronal activity^24^. The signal was amplified by 2000, band-passed from 0.5 to 100 Hz, using a 3 pole Butterworth filter, and sampled at 422 Hz by a 10-bit A/D converter (using ±2 V input range). The behavioral tasks and EEG data were synchronized with the LFP (PC + S) recordings using a short period (5–10 s) of low frequency stimulation pulse (5 Hz) at the beginning and end of each test.

Two types of recording were conducted: rest-state recordings and recordings during tasks.

#### Rest-state recordings

Participants were instructed to sit quietly for 3 min while bipolar recordings were obtained bilaterally for 30 s from each pair of electrodes (0–1, 0–2, 0–3, 1–2, 1–3, 2–3). Rest state recordings were obtained from OCD patients at the beginning and at the end of each session. Rest state recordings were obtained from PD patients at the beginning of each session while they were off medication (i.e., overnight cessation of all parkinsonian medications) and at the end of each session while they were on medication (i.e., at least an hour after taking their regular parkinsonian medications).

#### Recordings during tasks

Electrophysiological activity was recorded while subjects participated in four tasks:

##### The OCD provocative images task

In this task patients were exposed to a series of individually tailored OCD-relevant stimuli, aversive stimuli and neutral stimuli. Images were taken from a validated set of pictures, Berlin OCD – Picture Set (BOCD-PS), which was previously used by Simon et al^25^. As the ability to provoke OCD symptoms is culturally-dependent we validated a culture adjusted religious OCD provocation test in healthy Israeli volunteers. Prior to surgery, the OCD patients rated 250 pictures on a 0–10 scale according to the degree of their individual OCD arousal. A patient-specific OCD-relevant paradigm including neutral, aversive, or OCD provocative figures was built for each OCD patient. PD patients were presented with the paradigm of the OCD patient to whom they served as a control. During the recordings subjects were instructed to look at the screen in front of them. At every trial a figure appeared for 3 or 4 s, followed by a black screen presented for an intertrial interval (ITI) of one second.

##### The doubt task

This task was based on the hypothesis that a basic feature of OCD is patients’ difficulty to deal with daily doubts. Subjects were instructed to look at a screen in front of them. Each trial started with a 2 s presentation of a predictive cue which could have been a plus sign, a minus sign, or a question mark, followed by 1.5 s presentation of an image from the Geneva Affective Picture Database (GAPED)^26^. Plus and minus signs predicted the type of the following image, neutral or aversive, respectively, with 100% certainty. A question mark was followed with either an aversive or a neutral Image with equal probability.

##### The auditory Go-NoGo task

In this task a series of 120 tones, composed of frequent (82%) high pitch (1200 Hz) tone and deviant (18%) low pitch (300 Hz) tone were played in a pseudo-random order. The tone duration was 250 ms followed by 1000 ms ITI. Participants were instructed to press a handed button as fast as possible after the frequent “go” tone and to avoid pressing the button after the deviant “no go” tone. Subjects response was classified to four groups: “hit”—pressing the button in go trials, “miss”—not pressing the button in go trials, “commission error” (CE)—pressing the button in no-go trials, and “correct rejection” (CR)—not pressing the button in no-go trials.

##### The emotional voice recognition task

In this task subjects listened to and were asked to mentally recognize pseudo-randomized set of emotional voices from the Montreal affective voices (MAV) database, a validated database which consists of negative, neutral, and positive non-verbal male and female voices^27^.

In each recording session patients participated in all four tasks while bipolar recordings were obtained bilaterally for about 20–25 min from one pair of electrodes (either a ventral (0–2) or a dorsal (1–3) pair of electrodes). Task order was as follows: first, the OCD provocative images task; second, the doubt task; third, the Go-NoGo task; forth, the emotional voice recognition task. Each task lasted 3–5.5 min and the interval between tasks was 2–15 min.

### Stimulation parameters and medications

Monopolar review was conducted two weeks after surgery. Each contact was tested separately for the boundaries of the therapeutic window. Stimulation pulse duration was 60 μs with frequency and voltage adjusted to the individual patient. The therapeutic contact/s and configuration of contacts were selected according to the best immediate clinical effect together with the intraoperative data and anatomical localization of each contact. The OCD patients’ medical treatment remained stable with small changes in benzodiazepine medication dosage. The PD patients’ dopamine replacement treatment was reduced by ~50–75% according to their clinical state and the recommendation in the literature.

### Data analysis

#### Artifacts removal

In some of the recordings (mainly those containing the most ventro-medial contact) an electrocardiogram (ECG) artifact contaminated the neural activity signal. ECG pulses were identified by their high peak (above 1.2 SD over the mean) and regularity (CV < 0.22). In order to clean the data we adopted a template subtraction approach. We averaged all occurrences of the ECG signal in each recording to create a template, and then subtracted that template from every occurrence of the ECG artifact using linear regression to achieve optimal fit with the data. The efficiency of this method was evaluated by calculating the area under the spectrum and demanding its value to be below a visually set threshold. The presented data was recorded while stimulation was turned off, so no stimulation artifacts were recorded.

#### Spectral analysis

Data was band-pass filtered between 0.5 and 100 Hz using 4 poles Butterworth filter. Rest PSD was calculated using the Welch method with hamming window of 1 s and 50% overlap, and frequency resolution of 0.5 Hz. 50 Hz noise was extracted from the PSD and replaced with estimated values using Piecewise Cubic Hermite interpolation (PCHIP). Spectrum was than normalized by dividing it by its total power. For presentation purposes we flatten the spectrum by 1/f normalization (P′(f) = P(f)*f; (P′,P-original, normalized power, f-frequency). In order to compare STN rest spectral activity in OCD, PD on medication and PD off medication groups we calculated for each patient the PSD of the first five rest recording sessions starting a week after the surgery. We performed on these PSDs a one way ANOVA test for every frequency in the range of 3–80 Hz with 0.5 Hz resolution, and used Bonferroni correction to control for multiple comparisons. According to the results of this test we chose our frequencies of interest for further analysis to be theta: 6.5–8 Hz and high beta: 25–35 Hz.

#### Y-BOCS and theta/high beta correlations

**Fig. 1:**
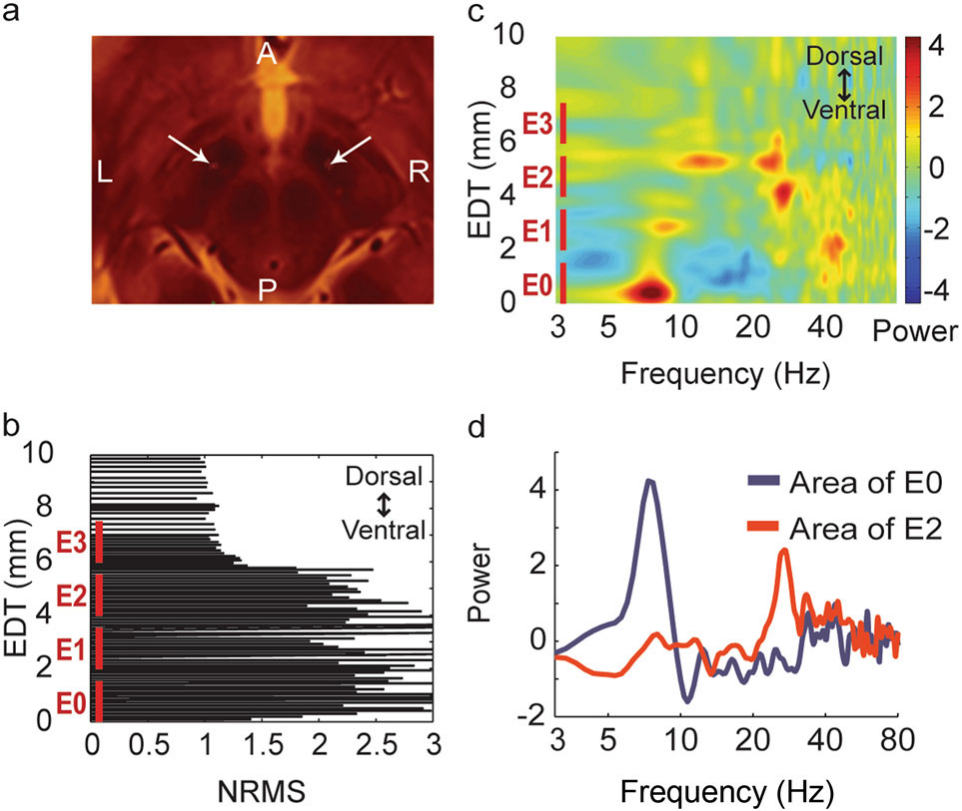
Intra-operative recordings of theta activity in ventro-medial STN in OCD patients. An example of intra-operative trajectory: **a** Electrodes location marked (white arrows) on reconstructions of the postop CT with the pre-op MRI of one OCD patient. **b**–**d** The normalized root mean squared (NRMS, **b**), power spectral density (PSD, **c**) and average PSD at contacts location (**d**) of one OCD patient right STN. PSD in **c** was smoothed with 2D Gaussian window of size 13 and standard deviation equal to 2. Window size is equivalent to 4 Hz on the *x* axis and 13 steps on the *y* axis. Step size varies according to location in the brain (see methods). EDT estimated distance to target (defined as STN center according to preoperative imaging)

One of our OCD patients, Jur02, participated in the study for over a year, during which her clinical state varied substantially. That allowed us to test for possible correlation between OCD clinical state and STN neuronal activity changes within the same patient. Based on the results of spectral activity in this patient (Fig. 1) we decided to focus on theta (6.5–8 Hz) and high beta (25–35 Hz) power recorded from the left and right ventral (E0–E2 and E8–E10 contacts) and dorsal (E1–E3 and E9–E11 contacts) STN. Power at a specific frequency range was defined as the difference between the peak value at that band and the closest minima. If there was no local maximum point at the frequency band the power value was set to zero. In days with more than one rest recording sessions, we averaged results of all sessions so that every day contributed one data point to the correlation analysis. Finally, a Spearman’s rank-order correlation was run to determine the relationship between clinical state and ventral STN theta and high beta power.

#### Tasks analysis

Data from right and left ventral (E0–E2 and E8–E10 contacts) and dorsal (E1–E3 and E9–E11 contacts) recording sites were included in the analysis. Single trials were extracted, and time frequency analysis was carried out using complex Morlet wavelet transform with a wavelet constant of 12 and center frequency bins ranging from 6.5 to 8 Hz. The result scalograms were corrected by subtraction of the average power at rest for each frequency and averaged above frequencies. Statistical analysis was carried out on averaged theta activity during time periods that were determined according to manual detection of response to task events. In the go-no go task statistical analysis was carried out on averaged theta activity during 0.4–1 s after the cue. In OCD provocative images task, the time-point for statistical analysis was 1–1.5 s after image presentation. In the doubt task the time-point for statistical analysis was 0.5–2 s after cue presentation. In emotional voices task the time-point for analysis was 0–1 s after voice onset. Noisy traces were identified visually and excluded from further analysis and outliers with z score higher than 2 were excluded. We conducted mixed ANOVA test with two factors: group (3: OCD, PD on medication, PD off medication) X condition (repeated measure factor, 3: task conditions). In case of a significant effect we conducted simple main effects procedure with Bonferroni correction for multiple comparisons.

#### Statistics

A threshold of 0.05 was used to establish statistical significance. All statistical tests were two-tailed. Data analyses were performed using Matlab 2013b (Mathworks, MA, USA) and statistical analyses were performed using SPSS 24.0 (IBM SPSS Statistics for Windows. Armonk, NY: IBM Corp).

## Results

**Fig. 2:**
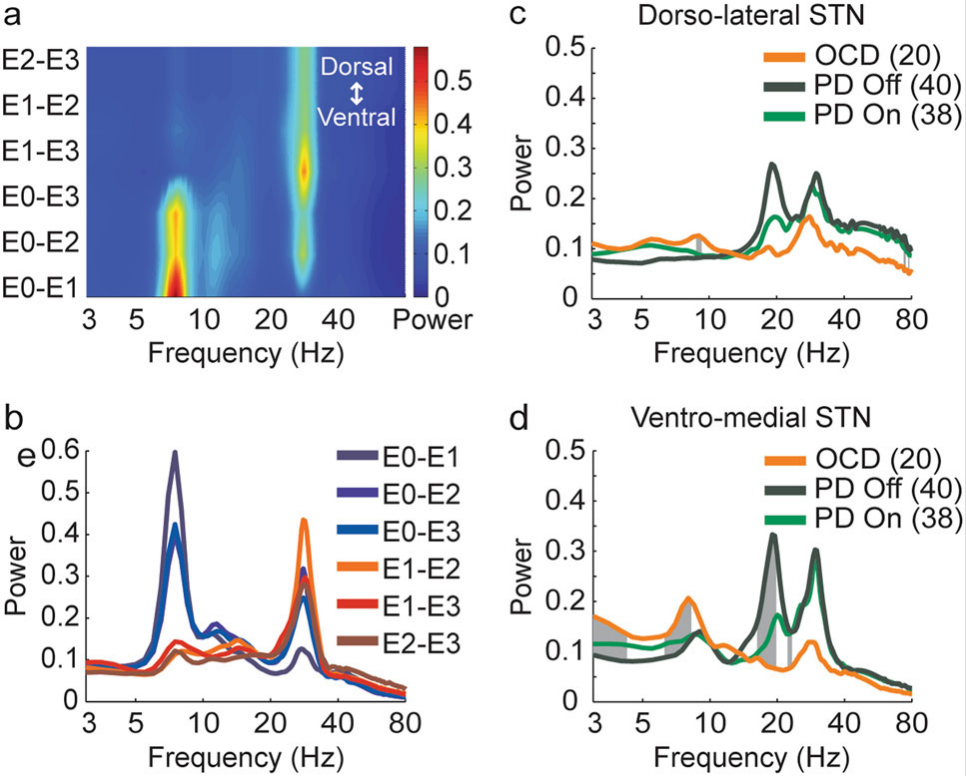
Post-operative recordings of beta and theta activity in dorso-lateral and ventro-medial STN in OCD and PD patients. **a**, **b** Average PSD (N = 52) of post-operative recordings of one OCD patient (Same patient as in Fig. 1), arranged spatially (**a**) and in a spectrogram (**b**). **c**, **d** Average spectral activity from dorsal (**c**; bipolar pairs: E2–E3 and E10–E11) and ventral (**d**; bipolar pairs: E0–E2 and E8–E10) STN of OCD and PD (on and off medication) patients in five recording sessions from one week to three months post operation. Gray areas indicate frequencies in which the difference between the groups is significant (*p* < 0.05 after Bonferroni correction)

Intraoperative microelectrode spiking activity recordings revealed distinct theta-oscillatory activity in the ventro-medial STN of OCD patients (Fig. 1a–d). Beta-oscillatory activity was identified in the dorso-lateral region of the STN in Parkinson’s patients and most interestingly also in OCD patients (Fig. 1b–d). Multiple macroelectrode LFP recordings during the first postoperative year clearly demonstrated that OCD patients had beta-oscillatory activity mainly at the dorso-lateral STN and theta-oscillatory activity mainly at the ventro-medial STN (Fig. 2a, b). Comparison of spectral activity from dorsal and ventral STN revealed significant differences in the theta and beta band frequencies between OCD and PD (on and off medication) patients (Fig. 2c, d).

**Fig. 3:**
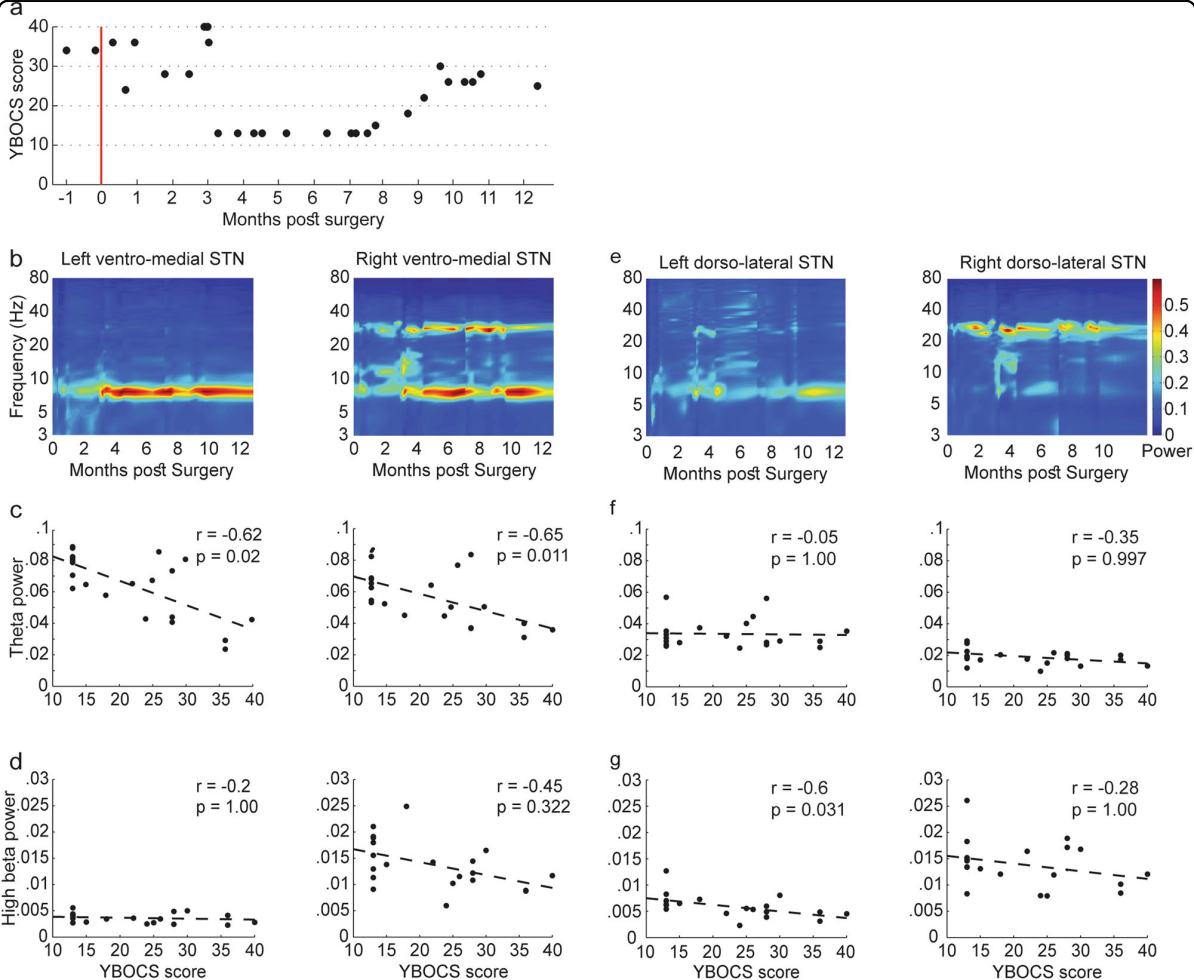
Theta activity in ventro-medial STN in OCD patients oppositely correlates with clinical symptoms. **a** Y-BOCS scores of one OCD patient (same as in Fig. 1) during one year post operation. Time point zero, marked with a red line, is the surgery day. **b** Left and right ventro-medial STN activity as a function of time post operation. Time axis is the same as in **a**. Spectral power was flattened by correction to 1/*f* (see methods). **c** Correlation of Y-BOCS scores with theta (6.5–8 Hz) activity in left and right ventro-medial STN. **d** Correlation of Y-BOCS scores with high beta (25–35 Hz) activity in left and right ventro-medial STN. **e** Left and right dorso-medial STN activity as a function of time post operation. Time axis is the same as in **a**. **f** Correlation of Y-BOCS scores with dorsal theta (6.5–8 Hz) activity in left and right dorso-medial STN. **g** Correlation of Y-BOCS scores with dorsal high beta (25–35 Hz) activity in left and right dorso-medial STN. Statistical results were obtained using Spearman’s rank-order correlation and Bonferroni correction for multiple comparisons

The first OCD patient’s clinical state was closely monitored and rated with the Yale-Brown Obsessive-Compulsive Scale (Y-BOCS) for the first postoperative year (Fig. 3a). As the OCD symptoms decreased (lower Y-BOCS score) the theta activity at the ventro-medial STN increased (Fig. 3b, c). After 8 months of treatment we observed an unexplained increase in the patient’s OCD symptoms severity that was accompanied by a decline in theta power. After another few weeks as the OCD symptoms decreased again, the theta activity increased. The severity of OCD symptoms was inversely correlated with the theta activity at the ventro-medial right (*r*_s_ = −0.65, *p* = 0.01) and left (*r*_s_ = −0.62, *p* = 0.02) STN, but not with high beta activity in those areas (Fig. 3c, d). The severity of OCD symptoms was also inversely correlated with high beta activity in the dorso-lateral left STN (*r*_s_ = −0.6, *p* = 0.03), but not right (*r*_s_ = −0.28, *p* = 1) dorso-medial STN (Fig. 3e–g). Theta activity in the dorso-medial STN did not correlate with Y-BOCS scores (Fig. 3f).

**Fig. 4:**
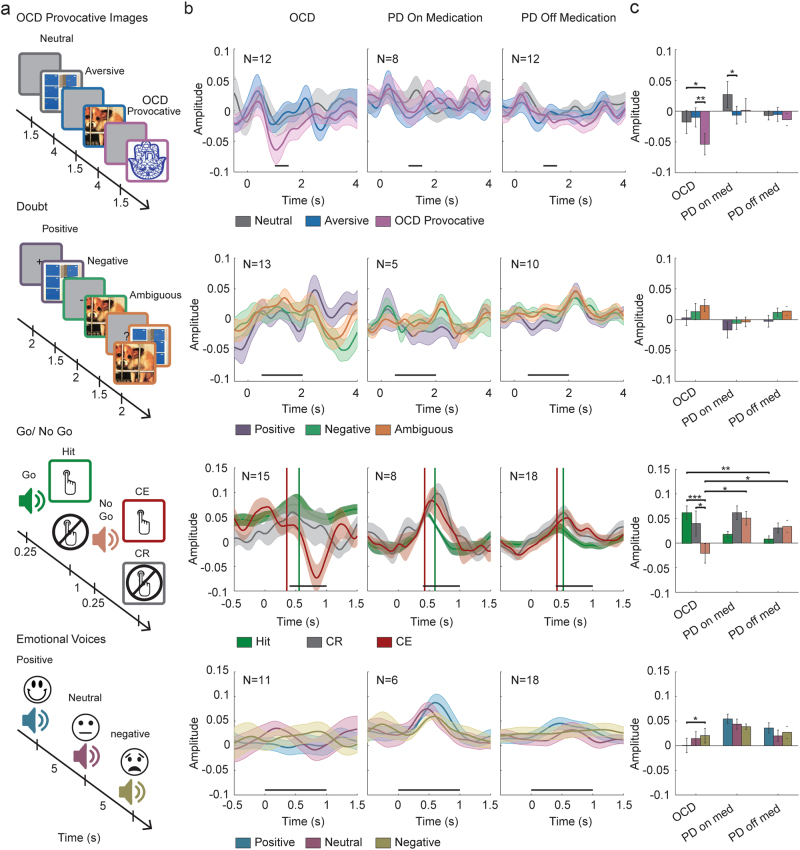
Functionality of ventro-medial STN theta activity in OCD vs PD patients. Patients were tested in four tasks **a** while STN activity was recorded: OCD provocative images task (neutral, aversive, and OCD provocative images); doubt task (positive, negative, and ambiguous cues); Go-NoGo task (hit, correct rejection (CR), and commission error (CE)); emotional voices task (positive, neutral, and negative voices). **b** Average ventro-medial STN theta power during tasks events. Shadows represent standard error of the mean. Time zero indicates trial beginning; black horizontal lines indicate time for statistical analysis. In the Go-NoGo task (third row) vertical lines indicate response time in hit (green) and CE (red) trials. **c** Average theta power during time selected for statistical comparison. Error bars represent standard error of the mean. *p < 0.05, **p < 0.01, ***p < 0.001

Behavioral tasks, including visual OCD provocation and doubt tests, auditory cognitive Go-NoGo paradigm and emotional voices recognition test downregulated ventro-medial STN theta activity in OCD patients but not in PD patients (Fig. 4a). OCD patients responded to OCD provocative images, commission errors in the Go-NoGo test and positive emotional voices in a decrease in theta activity. However, no OCD-specific responses in theta-band activity were found in doubt test and in response to emotional voices.

### OCD provocative task (Fig. 4b, c upper row)

Significant group × image interaction (F(4,58) = 3.653, *p* = .010), as well as significant main effect of images (F(2,58) = 6.456, *p* = .003) were found in the visual OCD provocation test. Post hoc test revealed that OCD patients responded to OCD provocative images in a decrease in theta activity (−.054 ± .014) and that response was more robust than their response to neutral (−010 ± .013, *p* = .0001) and aversive (−018 ± .015, *p* = .011) images. In addition a significant difference between PD patients on medication response to neutral (.027 ± .018) and aversive (−.007 ± .016, *p* = .045) images was found.

### Doubt task (Fig. 4b, c second row)

A main effect of cue type (F(2,50) = 3.464, *p* = .039) with no interaction effect was found in the visual doubt test. Post hoc tests showed that across all groups, activity after an ambiguous cue (.011 ± .006) was higher than after positive cue (−.006 ± .008, *p* = .029).

### Go-NoGo task (Fig. 4b, c third row)

A significant group × trial interaction (F(4,76) = 6.943, *p* = .00008) was found in the Go-NoGo paradigm. Post hoc tests showed that in OCD patients response to tone in commission error trials (−021 ± .016) was lower than in hit (.061 ± .01, *p* = .0001) and correct rejection trials (.04 ± .018, *p* = .013). OCD response to tone in hit trial was significantly higher than that of PD off medication (.007 ± .01, *p* = .002), and OCD response to tone in commission error trials was negative and opposite to PD response on (.051 ± .021, *p* = .031) and off medication (.034 ± .014, *p* = .041). Vertical lines indicate response time in hit (green) and CE (red) trials.

### Emotional voices task (Fig. 4b, c forth row)

A significant group x voice interaction (F(4,64) = 2.858, *p* = .030) was found in the emotional voices recognition test. Post hoc tests showed that OCD patients’ theta activity after positive voices (.001 ± .013) was significantly lower than after negative cue (.021 ± .014, *p* = .026). No significant difference was found in PD patients’ responses to the voices.

No significant interaction effects of patient condition (OCD or PD) and task events were found in dorsal activity in the OCD provocation test, the doubt test and the emotional voices recognition test (Supplementary Fig. 1). In the Go-NoGo paradigm a main effect of trial type (F(2,84) = 10.533, *p* = .00008) was found, with no interaction with group factor (Supplementary Fig. 1a-b third row).

## Discussion

Our results are based on two methodological advantages. First, we used the investigational device, Activa PC + S (Medtronic Inc.), that enables long-term LFPs recording and a correlation between STN activity and patient’s clinical state. Second, although OCD patients cannot be compared to healthy controls we used a matching control group of PD patients. In this study we have found a subcortical mechanism for OCD. First, the dorsal (motor) area of the STN in OCD patients displays a beta (25–35 Hz) oscillatory activity similar to PD patients whereas the ventral (limbic-cognitive) area of the STN displays distinct theta (6.5–8 Hz) oscillatory activity only in OCD patients. Second, these STN theta oscillations decrease with provocation of OCD symptoms and are inversely correlated with symptoms severity over time.

Our observations of high beta (25–35 Hz) oscillatory activity at dorso-lateral (motor) STN in OCD and PD patients challenge the concept that beta oscillations are pathognomonic for akinetic movement disorders. Beta oscillations have been suggested as a biomarker to trigger closed loop DBS for PD^28–30^. These results suggest a reconsideration of the optimal characteristics of these oscillations (in the temporal and/or frequency domains) for adaptive DBS paradigms^31^.

Another major finding is the unique theta oscillations recorded in the ventro-medial STN in OCD patients, correlated with the severity of OCD symptoms and modulated by provocation of OCD symptoms and other emotional and cognitive tasks. Ventro-medial STN theta activity might represent the level of inhibition of OCD symptoms: theta activity increases with successful inhibition of OCD symptoms and decreases with the failure to inhibit OCD symptoms. Previous intraoperative recordings of STN neurons in OCD patients have demonstrated a low firing rate and increased delta (1–4 Hz) activity but have not shown a correlation between OCD severity and mean discharge rate^19,20^. OCD symptoms were related to changes in different frequency bands, with no clear preferential oscillatory pattern in postoperative recordings from two patients during unexpected provocation of OCD symptoms that occurred during a cognitive task^22^. STN activity during a decision-making task revealed that checking behavior was associated with a higher STN firing rate and that stopping decisions were associated with beta band activity^22,32^. Recordings from other DBS targets in OCD patients revealed high frequency discharge and variability of inter-spike intervals in the caudate nucleus (*n* = 2) and lack of alpha-band (8–14 Hz) oscillatory activity in the bed nucleus of stria terminalis (in 5 OCD patients vs. 7 major depression patients)^33,34^. In a recent study using combined LFP and magnetoencephalography (MEG) recordings, theta-coupling was evident between the STN and the anterior cingulate cortex^23^.

The differences in investigational methods might explain the variability in oscillatory activity between this study and the previous literature. All previous electrophysiological recordings in OCD patients were performed either intra-operatively or in the first post-operative days. Awake DBS surgery involves considerable amount of stress and anxiety that might alter neuronal activity. The short post-operative period might be confounded with micro-lesion, local edema and insertional effect that impact the electrophysiological activity. Our results obtained in a neutral outpatient set-up over a long period of clinical follow-up emphasize the importance of theta oscillation in the physiology of OCD symptoms. Nevertheless, our study is limited by a small number of OCD patients and further verification of these results in a larger sample of patients is needed.

Few studies have correlated STN low frequency oscillations (theta (4–8 Hz) and alpha (8–13 Hz) frequency bands) with motor, cognitive, and emotional clinical effects. STN low frequency (4–10 Hz) oscillations were correlated with levodopa-included dyskinesia and impulse control disorders in PD patients^35,36^. Presentation of emotional stimuli has been shown to reduce STN alpha oscillatory activity in PD patients^37–39^. High-conflict tasks has been shown to increase STN theta oscillatory activity^40–43^ and conflictual moral stimuli have been shown to increase STN theta and alpha (5–13 Hz) oscillatory activity^44^. Slowing the responses in a simple response-inhibition task has been correlated with increased STN alpha oscillatory activity^45^.

Our results are in line with these previous reports, showing a correlation between STN theta oscillatory activity and response inhibition. We show an increased theta oscillatory activity during response and response-inhibition in the Go-NoGo task both in PD and OCD patients (Fig. 4 third row). However, failure to inhibit responses (commission errors) is correlated with a decreased theta oscillatory activity in OCD patients. Similarly, provocation of OC symptoms (OCD provocation task, Fig. 4 first row) is correlated with a decreased theta oscillatory activity in OCD patients. Both commission errors and OC symptoms are a failure to inhibit the urge to inappropriate thoughts or behaviors and are correlated with decreased STN theta activity. Long-term successful inhibition of the urge to inappropriate thoughts or behaviors is clinically represented by the decreased severity of OCD symptoms (decreased Y-BOCS score) and is correlated with increased theta oscillatory activity (Fig. 3).

It is worth noting that comparing OCD and PD patients has some limitation related to the different diagnosis, age, duration of disease, comorbid illness, medication intake, and so on. Another possible difference between the OCD and PD patients is the site of electrode implantation. In OCD patients the electrodes were 2 mm anterior to and 1 mm medial to the target that was used for PD patients. Analysis of the intra-operative recordings of STN in both OCD and PD patients enabled identification of the beta oscillatory activity and differentiation of the dorso-lateral area from the ventro-medial area of the STN for each patient (see an example in Fig. 1b–d).

In conclusion, we show that STN theta oscillatory activity can modulate cognitive and emotional aspects of OCD. We suggest that theta activity (or decreased theta activity) at the ventro-medial STN might be used as a biomarker for closed-loop feedback-controlled stimulation to treat OCD: specific stimulation that enhances theta activity might reduce the severity of OCD symptoms.

## Electronic supplementary material

Supplementary Data

Supplementary Figure 1
