# Supplementary material for: Subthalamic theta activity: a novel human subcortical biomarker for obsessive compulsive disorder
Source: Transl Psychiatry. 2018 Jun 18;8:118. doi: 10.1038/s41398-018-0165-z (PMC6006433; doi:10.1038/s41398-018-0165-z)
Supplement: Supplementary file 1 — Supplementary Data [file 41398_2018_165_MOESM1_ESM.docx]

**SUPPLEMENTARY DATA:**

**SUPPLEMENTARY FIGURE LEGEND:**

**Supplementary Figure 1: Functionality of dorso-lateral STN theta activity in OCD vs PD patients**

Patients were tested in four tasks while STN activity was recorded as detailed in figure 4. (A) Average ventral STN theta power during tasks. Shadows represent standard error of the mean. Time zero indicates trial beginning; black horizontal lines indicate time for statistical analysis. In the Go-NoGo task (third row) vertical lines indicate response time in hit (green) and CE (red) trials. (B) Average theta power during time selected for statistical comparison. Error bars represent standard error of the mean.
