# Supplementary figures and images for: Subthalamic theta activity: a novel human subcortical biomarker for obsessive compulsive disorder
Source: Transl Psychiatry. 2018 Jun 18;8:118. doi: 10.1038/s41398-018-0165-z (PMC6006433; doi:10.1038/s41398-018-0165-z)

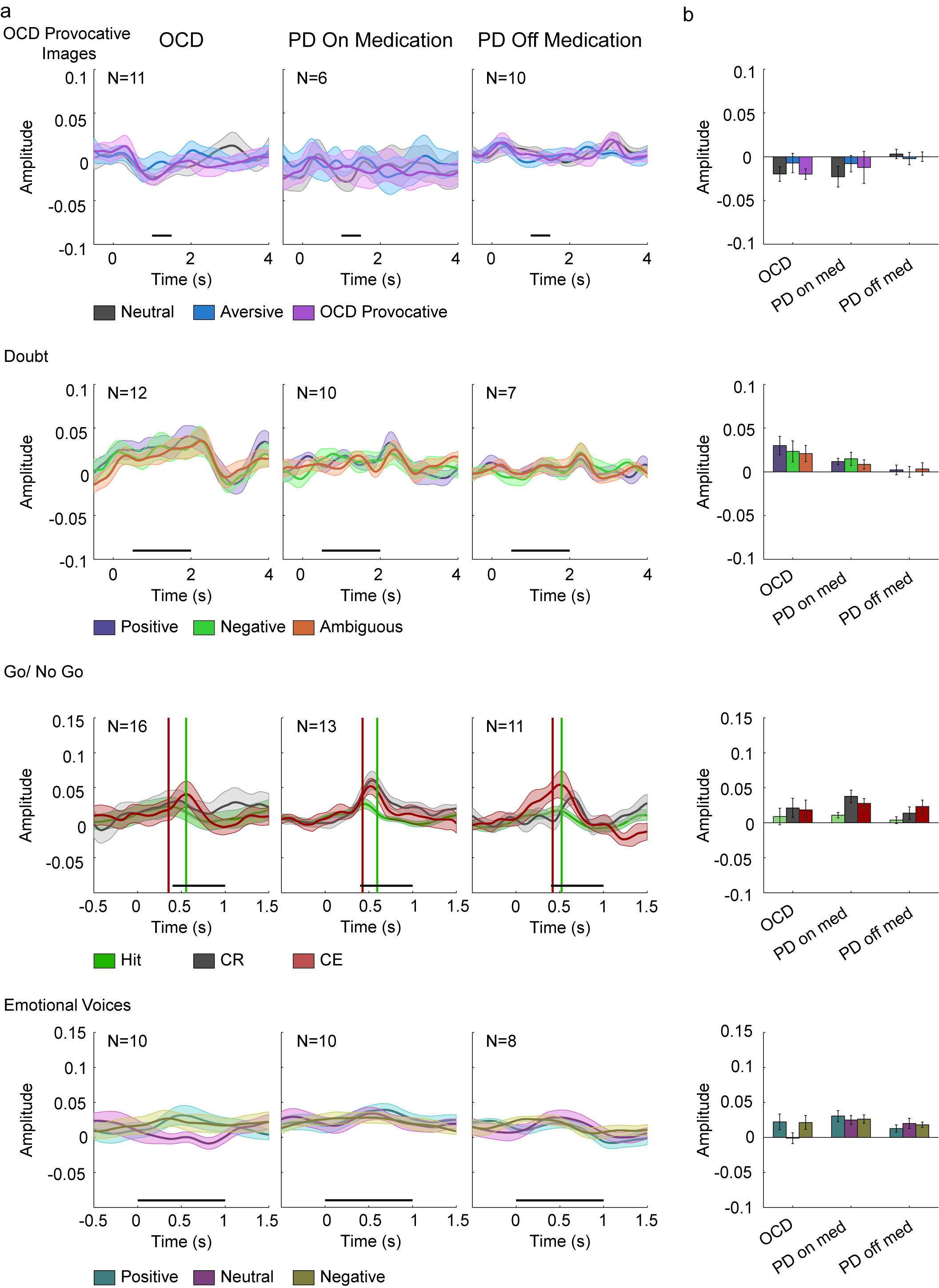

Supplement: Supplementary file 2 — Supplementary Figure 1 [file 41398_2018_165_MOESM2_ESM.tif]
